# Profiling and engineering of microRNAs for enhancing recombinant protein productivity in Chinese hamster ovary cells

**DOI:** 10.1186/1753-6561-7-S6-P102

**Published:** 2013-12-04

**Authors:** Wan Ping Loh, Bernard Loo, Lihan Zhou, Peiqing Zhang, Dong Yup Lee, Yuan Sheng Yang, Kong Peng Lam

**Affiliations:** 1 Bioprocessing Technology Institute, 20 Biopolis Way, #06-01 Centros, Singapore 138668; 2 Department of Biochemistry, National University of Singapore, 8 Medical Drive 4, Blk MD7 #05-04, Singapore 117597

## Background

Chinese hamster ovary (CHO) cells have become dominant host cells in the biopharmaceutical industry due to their capacity for proper protein folding, assembly and post-translational modifications. However, low specific productivity (qp) places limitations on yields obtained from mammalian host cells. MicroRNAs (miRNAs), a novel class of short, non-coding RNAs which negatively regulate target gene expression at post-transcriptional levels, have emerged as promising targets for engineering of CHO cell factories to enhance recombinant protein production. While engineering of miRNAs for enhanced cell growth and delayed cell death have been reported, miRNA targets which can enhance qp have not been identified to date.

## Materials and methods

To understand the role of miRNAs in conferring high qp phenotype in CHO cells, we carried out high throughput sequencing of 4 in-house generated IgG-expressing CHO sub-clones of varying qps. Reads were mapped to miRBase and 22 miRNAs were found to be differentially expressed between the high and low producers. These miRNAs were stably transfected into an IgG-expressing sub-clone to assess their effects on growth, titer, qp and product quality attributes.

## Results

Over-expression of miRs-17, 19b, 20a and 92a individually and in combination resulted in 13-27% increases in titer and 14-24% increases in qp in stably transfected pools. No significant alterations in proliferation rates were observed. 20-45 single cell clones were randomly selected from each of the 5 transfected pools for characterization. Statistical analyses showed significant differences in titer/qp between the high- and low-miRNA expressing single cell clones. The highest producing single cell clones exhibited ~100% increases in titer and qp compared to non-transfected cells. A correlation was found between increased miR-19b levels (>1.3-fold) and enhanced qp and titer. Over-expression of miR-19b does not appear to impact IgG aggregation significantly.

## Conclusions

To our knowledge, this is the first report of enhancement of recombinant protein productivity by stable miRNA over-expression. The genes and cellular pathways targeted by these miRNAs specific to enhancing protein productivity are under investigation and will be reported.

